# Supplementary material for: The interactome of intact mitochondria by cross-linking mass spectrometry provides evidence for coexisting respiratory supercomplexes
Source: Mol Cell Proteomics. 2017 Dec 8;17(2):216–32. doi: 10.1074/mcp.RA117.000470 (PMC5795388; doi:10.1074/mcp.RA117.000470)
Supplement: Supplemental Data [file supp_RA117.000470_133922_0_supp_23978_fzffwf.zip › spectra_annotation/mito_DR_spectra_annotation/379-1-8-1-19-1.pdf]

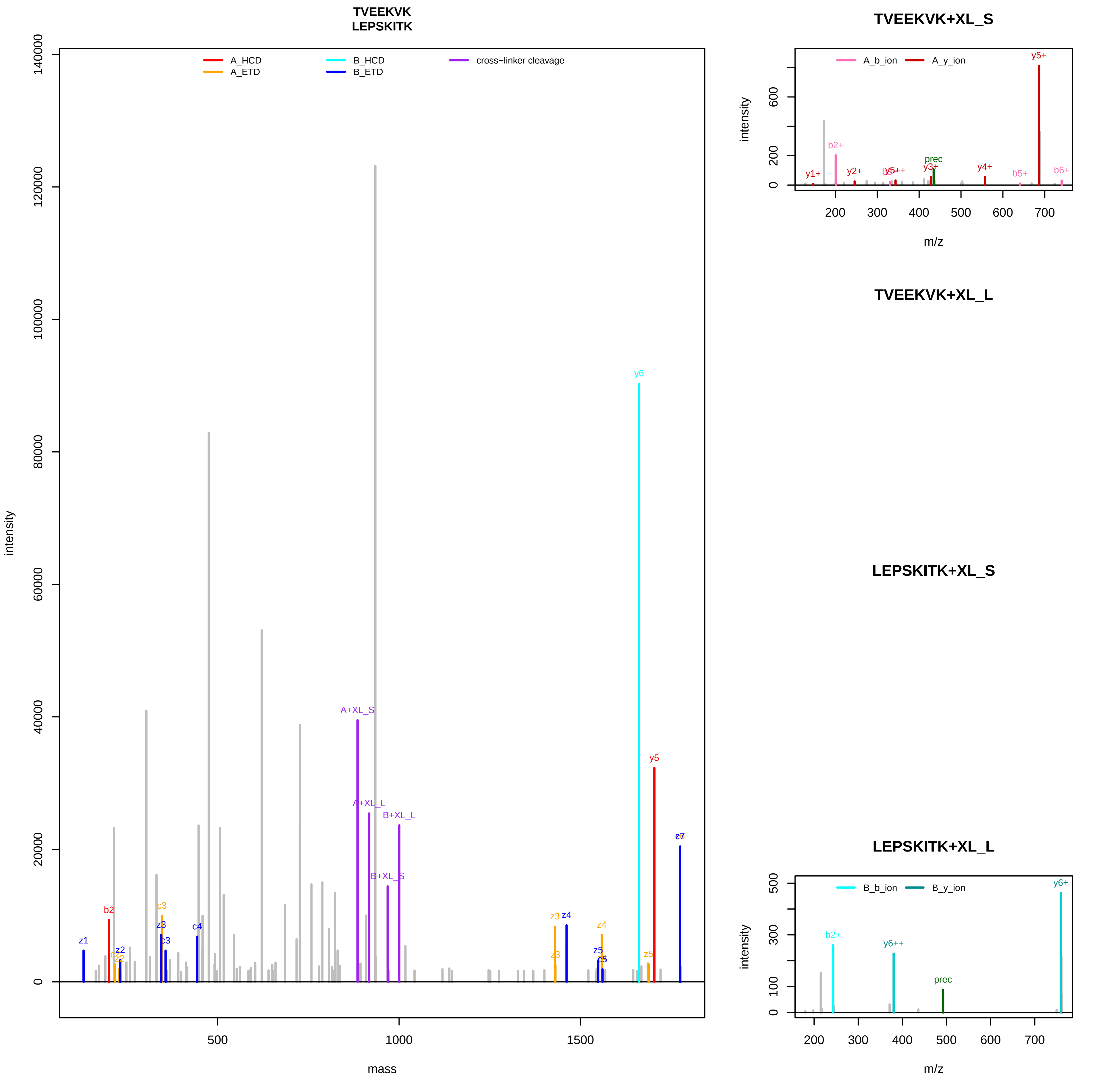
TVEEKVK
LEPSKITK
A_HCD
A_ETD
B_HCD
B_ETD
cross−linker cleavage
intensity
mass
0
20000
40000
60000
80000
100000
120000
140000
500
1000
1500
z1
b2
c2
z2
c3
z3
c3
c4
A+XL_S
A+XL_L
B+XL_L
B+XL_S
z3
z3
z4
z4
z5
c5
y6
y5
z5
c7
TVEEKVK+XL_S
A_b_ion
A_y_ion
y5+
b2+
prec
y1+
y2+
y5++
y3+
y4+
b5+
b6+
intensity
m/z
0
200
600
200
300
400
500
600
700
TVEEKVK+XL_L
LEPSKITK+XL_S
LEPSKITK+XL_L
B_b_ion
B_y_ion
y6+
b2+
y6++
prec
intensity
m/z
0
100
300
500
200
300
400
500
600
700
